# Supplementary material for: Low-cost 3D-printed optics for super-resolution multifocal structured illumination microscopy
Source: Biomed Opt Express. 2026 Jan 13;17(2):769–83. doi: 10.1364/BOE.583760 (PMC12904558; doi:10.1364/BOE.583760)
Supplement: Supplement 1 [file boe-17-2-769-s001.pdf]

Supplemental Document

Biomedical Optics EXPRESS

Low-cost 3D-printed optics for super-resolution multifocal structured illumination microscopy: supplement

JAY CHRISTOPHER,[1,*] LIAM M. ROONEY,[2,3] CHARLIE BUTTERWORTH,[1] GAIL MCCONNELL,[3] AND RALF BAUER[1]

[1] *Electronic and Electrical Engineering, University of Strathclyde, Glasgow, UK*
[2] *School of Infection & Immunity, University of Glasgow, Glasgow, UK*
[3] *Strathclyde Institute of Pharmacy and Biomedical Sciences, University of Strathclyde, Glasgow, UK*
[*] *jay.christopher@strath.ac.uk*

This supplement published with Optica Publishing Group on 13 January 2026 by The Authors under the terms of the Creative Commons Attribution 4.0 License in the format provided by the authors and unedited. Further distribution of this work must maintain attribution to the author(s) and the published article's title, journal citation, and DOI.

Supplement DOI: https://doi.org/10.6084/m9.figshare.30857489

Parent Article DOI: https://doi.org/10.1364/BOE.583760

Low-Cost 3D Printed Optics for Super-Resolution Multifocal Structured Illumination Microscopy

JAY CHRISTOPHER[1,*], LIAM M. ROONEY[2,3], CHARLIE BUTTERWORTH[1], GAIL MCCONNELL[3], AND RALF BAUER[1]

[1] *Electronic and Electrical Engineering, University of Strathclyde, Glasgow, UK*
[2] *School of Infection & Immunity, University of Glasgow, Glasgow, UK*
[3] *Strathclyde Institute of Pharmacy and Biomedical Sciences, University of Strathclyde, Glasgow, UK*
*[*jay.christopher@strath.ac.uk](mailto:jay.christopher@strath.ac.uk)*

ORCID IDs: JC 0009-0009-0707-8947; LR 0000-0002-2237-501X; CB 0009-0000-0380-0642; GM 0000-0002-7213-0686; RB 0000-0001-7927-9435

FFTs of the Widefield, Laser-Scanned, and mSIM BPAE images

In this section we present the FFTs for each respective lenslet array and each respective imaging modality (widefield, laser-scanned with deconvolution, mSIM with deconvolution).

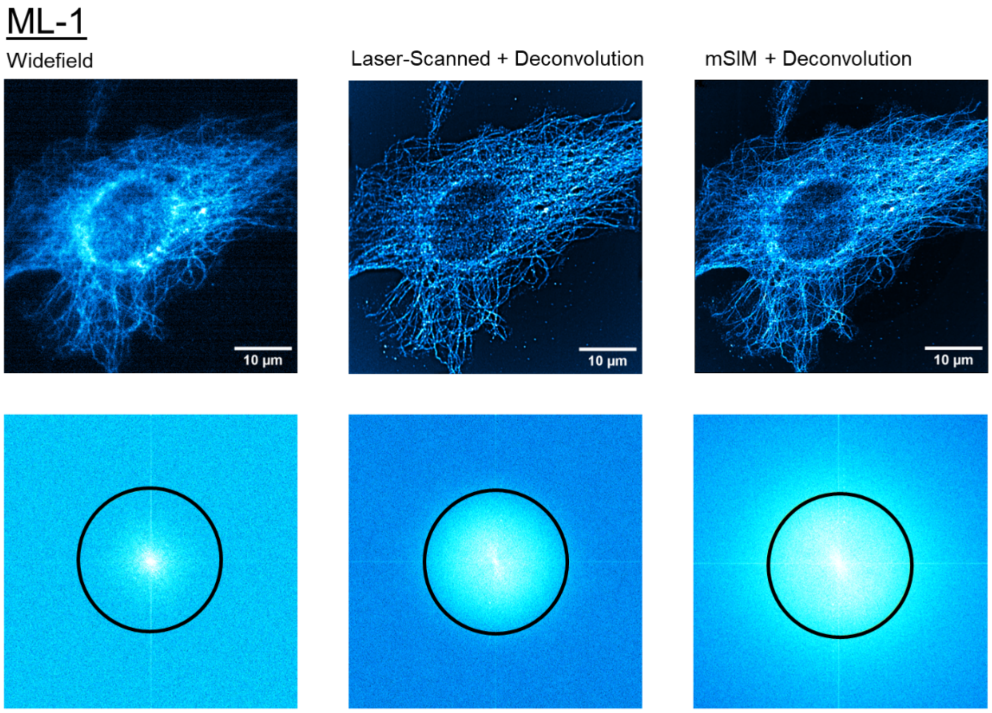

Fig. S1: BPAE widefield and maximum intensity projection images with each corresponding FFT for commercial array ML-1. Circular overlay ≈ 237 nm.

The BPAE images associated with lenslet array ML-1 are shown with their respective FFTs below each imaging modality. The BPAE images were contrast enhanced using FIJI exclusively for visual clarity to the reader. Each FFT image has the same circular overlay representing the resolution limit under laser-scanning with deconvolution conditions Figure S1

shows that high-frequency information surpasses the circular overlay in the mSIM + Deconvolution BPAE image, supporting the *a-Tubulin* line profile results in the manuscript.

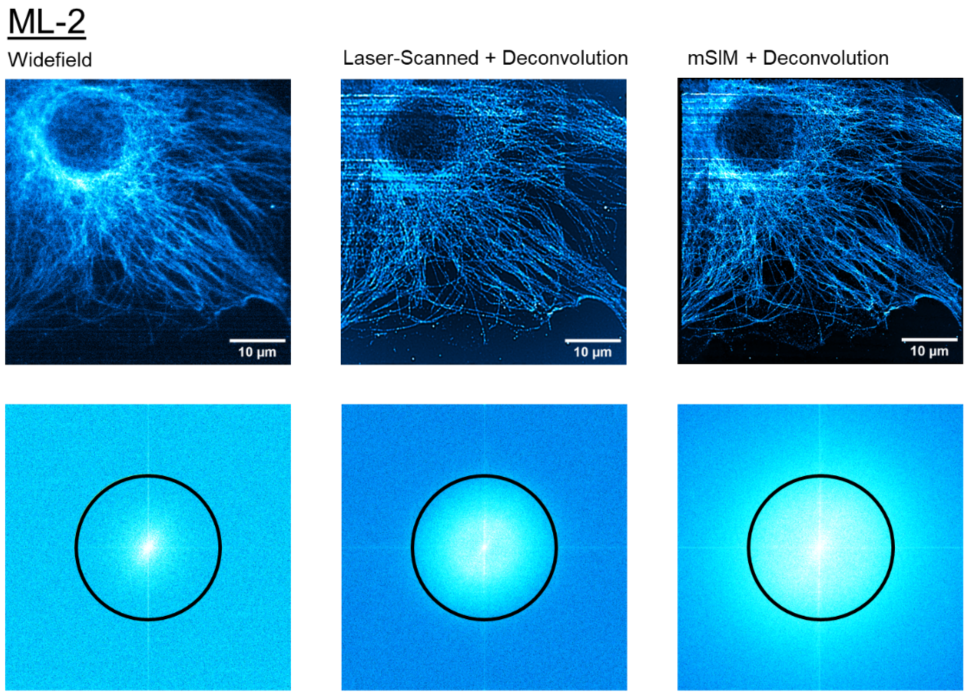

Fig. S2: BPAE widefield and maximum intensity projection images with each corresponding FFT for commercial array ML-2. Circular overlay ≈ 244 nm.

The BPAE images associated with lenslet array ML-2 are shown with their respective FFTs below each imaging modality. The BPAE images were contrast enhanced using FIJI exclusively for visual clarity to the reader. Each FFT image has the same circular overlay representing the resolution limit under laser-scanning with deconvolution conditions. Figure S2 shows that high-frequency information surpasses the circular overlay in the mSIM + Deconvolution BPAE image, supporting the *a-Tubulin* line profile results in the manuscript.

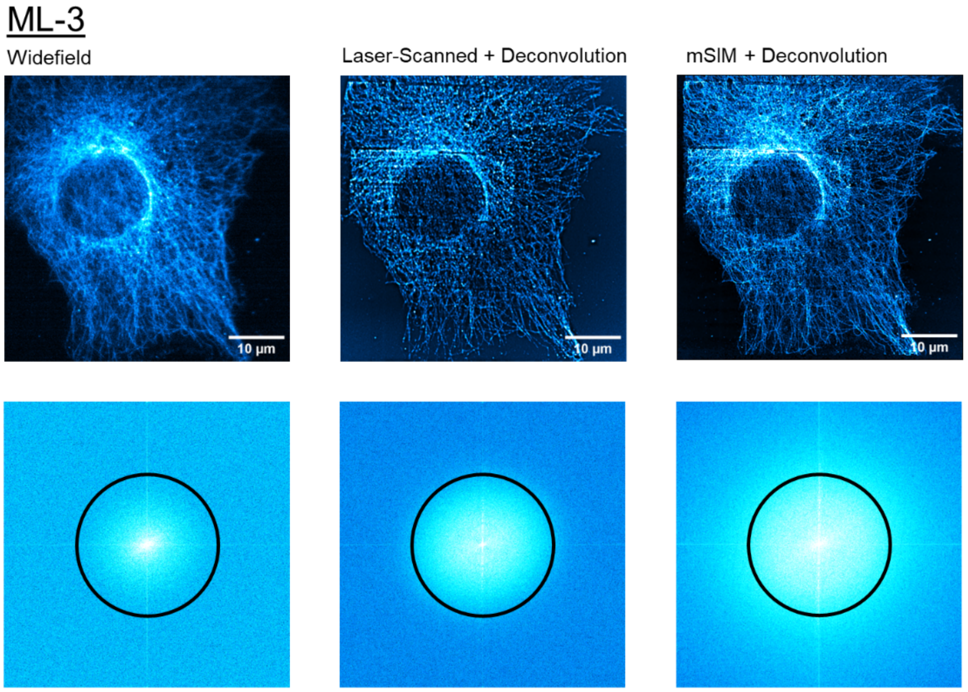

Fig. S3: BPAE widefield and maximum intensity projection images with each corresponding FFT for commercial array ML-3. Circular overlay ≈ 232 nm.

The BPAE images associated with lenslet array ML-3 are shown with their respective FFTs below each imaging modality. The BPAE images were contrast enhanced using FIJI exclusively for visual clarity to the reader. Each FFT image has the same circular overlay representing the resolution limit under laser-scanning with deconvolution conditions. Figure S3 shows that high-frequency information surpasses the circular overlay in the mSIM + Deconvolution BPAE image, supporting the *a-Tubulin* line profile results in the manuscript.
